# Sensitization to Drug Treatment in Precursor B-Cell Acute Lymphoblastic Leukemia Is Not Achieved by Stromal NF-κB Inhibition of Cell Adhesion but by Stromal PKC-Dependent Inhibition of ABC Transporters Activity

**DOI:** 10.3390/molecules26175366

**Published:** 2021-09-03

**Authors:** Paola Fernanda Ruiz-Aparicio, Gloria Inés Uribe, Adriana Linares-Ballesteros, Jean-Paul Vernot

**Affiliations:** 1 Grupo de Investigación Fisiología Celular y Molecular, Facultad de Medicina, Universidad Nacional de Colombia, Bogotá 111321, Colombia; pfruiza@unal.edu.co; 2 Servicio de Patología, Laboratorio de Hematología Especial y Citometría de Flujo, Fundación Hospital de la Misericordia, Bogotá 111071, Colombia; guribeb@homifundacion.org.co; 3 Grupo de Investigación Oncohematología Pediátrica, Fundación Hospital de la Misericordia, Universidad Nacional de Colombia, Bogotá 111071, Colombia; talinaresb@unal.edu.co; 4 Instituto de Investigaciones Biomédicas, Facultad de Medicina, Universidad Nacional de Colombia, Bogotá 111321, Colombia

**Keywords:** mesenchymal support, B-ALL, chemotherapy, PKC inhibition, chimeric peptide, NF-κB signalling, ABC transporters, cell-cell interaction, leukemic microenvironment

## Abstract

Cell adhesion to stromal support and the associated intracellular signaling are central to drug resistance, therefore blocking both has been effective in increasing drug sensitization in leukemia. The stromal Ser/Thr protein kinase C (PKC) has been found to be important for conferring protection to leukemic cells. We aimed at elucidating the intracellular signals connected to cell adhesion and to stromal PKC. We found that NF-κB and Akt were up-regulated in mesenchymal stem cells (MSC) after binding of B-cell acute lymphoblastic leukemia (B-ALL) cells. Nevertheless, Akt inhibition did not induce B-ALL cell detachment. In spite of a clear activation of the NF-κB signaling pathway after B-ALL cell binding (up-regulation NF-κB1/2, and down-regulation of the IKBε and IKBα inhibitors) and an important reduction in cell adhesion after NF-κB inhibition, sensitization to the drug treatment was not observed. This was opposite to the PKC inhibitors Enzastaurin and HKPS, a novel chimeric peptide inhibitor, that were able to increase sensitization to dexamethasone, methotrexate, and vincristine. PLCγ1, Erk1/2, and CREB appear to be related to PKC signaling and PKC effect on drug sensitization since they were contra-regulated by HKPS when compared to dexamethasone-treated cells. Additionally, PKC inhibition by HKPS, but not by Enzastaurin, in MSC reduced the activity of three ABC transporters in leukemic cells treated with dexamethasone, a new indirect mechanism to increase sensitization to drug treatment in B-ALL cells. Our results show the validity of targeting the functional characteristic acquired and modulated during cell-to-cell interactions occurring in the leukemic niche.

## 1. Introduction

Acute lymphoblastic leukemia (ALL) is a heterogeneous disease with diverse genetic alterations [1]. The survival rate has improved in the last decades, reaching 80–90% today. Nevertheless, cure rates, after patients relapse, are much lower [2]. In general, it is accepted that genetic diversity exists at both diagnosis and relapse and that these former leukemic clones derive from either major or minor clones at disease presentation [3]. Nevertheless, leukemic cells obtained from patients with early relapse may also have developed new mutations that hinder treatment and have a poor outcome [1]. In ALL, these cells have properties of long-term dormancy, treatment resistance, and interestingly also some stemness properties [4]. Of note, these therapeutically unfavorable features were partially reversed when cells were dissociated from the microenvironment. This suggests that ALL relapse may occur also due to protection and support by the bone marrow (BM) microenvironment in privileged niches [5,6]. In fact, anchoring to a remodeled BM leukemic niche that interferes with normal hematopoiesis [7,8,9,10] favoring leukemic growth is responsible for resistance to chemotherapy and disease relapse not only in ALL but also in other types of leukemia [11,12,13,14]. Therefore, targeting the leukemia-supportive niche to increase sensitization to drug therapy may be an additional strategy to halt disease progression [15,16,17]. 

Since the BM microenvironment is the main site of relapse in pediatric B-ALL [18], knowing the stromal cell players and the diverse mechanisms of cell protection is essential for the development of novel therapeutic strategies. The task is not straightforward, since the dynamic interactions between leukemic cells and the diverse BM niche components may oscillate between tailor-made niches in time and space [8] and more diffused and generalized protective mechanisms [19]. Additionally, these interactions are both ways with leukemic cells altering stromal cells and vice versa and between cells presenting higher heterogeneity [6]. In spite of that complexity, in a mouse model of ALL, it was shown that restoration of the hematopoietic function was a direct consequence of intervening the leukaemia-induced microenvironment rather than decreasing leukaemia burden [20]. 

It has been shown that mesenchymal stem cells (MSC), a main component of the BM niche, are responsible in part for this protective effect [21,22]. Cell interactions occurring through the CXCR4/CXCL12 chemokine axis and specific cell adhesion molecules, both inducing a strong binding of B-ALL cells to MSC, are supposed to be the main mediators of this protection [23,24]. In particular, CD44, VCAM, ICAM, LFA-1, selectins, galectins, and other integrins in MSC have been implicated [24,25,26,27]. Furthermore, therapy-resistant ALL cells have enriched expression of cell adhesion genes [4]. The activation of these adhesion molecules and their corresponding downstream signalling, which includes NF-κB, FAK, MAPK, PI3K/Akt, among others, account for leukemic cell survival and drug resistance [28,29]. Early work has shown that antibodies directed against these adhesive contacts or pharmacological inhibition of these interactions are effective in the reversal of the chemoresistance in several types of leukemia [30,31,32]. In addition, blocking specific signaling pathways in stromal cells (Erk, p38, PI3K/Akt, PKC, and others) through specific inhibitors has been useful for this purpose [29,33,34]. The use of pan or isoform specific PI3K inhibitors, alone or in combination with dexamethasone (DEXA), has been used to overcome glucocorticoid resistance in B-ALL [35]. Inhibitors of Akt and Erk in combination with Nilotinib have reduced the ability of ALL cells to develop resistance [36]. Furthermore, the inhibition of NF-κB signaling reversed the chemoprotective effect of BM niche cells exposed to tumor-targeting agents in a biomimetic model of B-ALL [37].

It was shown that the induction of murine or human stromal PKC-βII is a mechanism to ensure chronic lymphocytic leukemia (CLL) survival [38]. This effect was attributed to the up-regulation and secretion of the pro-inflammatory cytokines IL-1α and IL-15 by activation of the canonical NF-κB pathway downstream of PKC-βII activation. Interestingly, this was also true for other hematological malignancies, ALL, and mantle cell lymphoma, suggesting that it could be a generalized stromal protection mechanism in leukemia [38]. More recently, it was shown that stromal PKC inhibition, by a chimeric peptide HKPS or the conventional inhibitors Enzastaurin or Staurosporine, increased the chemosensitivity to drug treatment in B-ALL [16] and CLL [17]. In the first study, down-regulation of the adhesion molecules ICAM-1 and VLA-5 and the consequent inhibition of cell adhesion by PKC inhibitors was proposed as the mechanism to induce sensitization to dexamethasone treatment [16], while in the second study, stabilization of BCL-X_L_ and up-regulation of TFEB were suggested as the basis to overcome drug resistance [17]. 

Taking into consideration that many possible mechanisms exist that can be involved in drug resistance [37,39], and knowing that interactions between MSC and leukemia cells are diverse and complex, including cell-to-cell contacts, nanotunneling, exosomes, vesicles, and soluble factors [40,41,42], the aim of the present work was to gain a better understanding of the signaling molecules and mechanisms that could be involved in drug resistance induced indirectly by MSC in childhood B-ALL.

## 2. Results

### 2.1. Increased Susceptibility to MTX and VNC with Inhibitors of Stromal PKC

**Figure 1:**
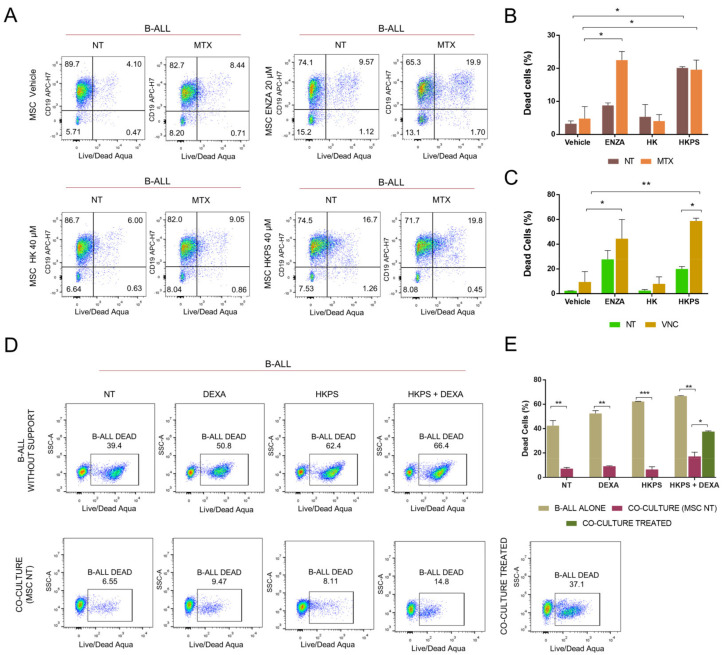
B-ALL cells show increased susceptibility to conventional chemotherapy after PKC inhibition in the mesenchymal support. (**A**) Cell viability of primary B-ALL cells after PKC inhibition and treatment with MTX. As control, B-ALL cells without treatment (NT) were included. MSC were treated with HKPS, the commercial inhibitor ENZA, or the controls for 2 h, as indicated. B-ALL cells, pre-treated 6 h with 6.25 µM of MTX, were co-cultured for additional 72 h on MSC. Co-cultures were collected after trypsinization and the cell viability was assessed by flow cytometry on the leukemic cell population through the double staining with the reactive LIVE/DEAD Aqua and the anti-CD19 antibody. A representative experiment is shown. (**B**) Quantification of the B-ALL cell population double positive for CD19 and LIVE/DEAD Aqua as described in A. (**C**) Quantification of the viability of the B-ALL cells after inhibition of PKC in MSC with HKPS, HK (40 µM), ENZA (20 µM), or the vehicle (0.4% DMSO) and co-culturing for 72 h with B-ALL cells pre-treated with VNC (500 nM). (**D**) Simultaneous effect of DEXA and HKPS on B-ALL cells viability. Leukemic cells were pre-treated for 6 h with DEXA (250 nM), HKPS (40 µM), or both, and then they were co-cultured for 24 h with non-treated MSC (CO-CULTURE (MSC NT)); also, co-cultures were established and then cells were treated with the combination of HKPS and DEXA (CO-CULTURE TREATED); as controls, B-ALL cells without support were employed (B-ALL ALONE, upper panel). Cell viability was assessed by flow cytometry. A representative experiment is shown. (**E**) Percentages of B-ALL cells viability in the conditions indicated above. Data are expressed as mean ± SEM (*p* values: Non-parametric one-way ANOVA. * *p* < 0.05. ** *p* < 0.01. *** *p* < 0.001) from two independent experiments.

The protective effect of MSC on leukemic cells and the role of PKC-β in this process were previously demonstrated in CLL [17]. We have also recently shown that the pre-treatment of MSC with typical and novel chimeric peptide PKC inhibitors, prior to the establishment of co-cultures, renders B-ALL cell lines and primary B-ALL cells more susceptible to treatment with dexamethasone (DEXA) [16]. Therefore, we first studied whether the increased susceptibility to DEXA could be observed with other frontline drugs used for B-ALL after MSC PKC inhibition (Figure 1). In fact, this was the case for methotrexate (MTX) with prior Enzastaurin (ENZA) treatment of MSC (Figure 1A,B) and for vincristine (VNC) when MSC were previously treated with the chimeric peptide HKPS or ENZA (Figure 1C). The vehicle or control peptide (HK) used in these experiments had no effect. Sensitization to VNC was higher than to MTX, with HKPS reaching more than 50% cell death. It should be emphasized that in these experiments, the B-ALL cells were not exposed to the PKC inhibitors used, showing solely their indirect effect over the mesenchymal support on B-ALL survival. Then we analyzed the effect of PKC inhibition together with DEXA treatment, in both B-ALL cells and co-cultures. First, treatment of B-ALL cells with DEXA, HKPS, or both induced more cell death (51–66%) than the control non-treated B-ALL cells (39%) (Figure 1D). As expected, this cytotoxic effect was markedly diminished (6–15%) by incubation with MSC (Figure 1E); nevertheless, if co-cultures were simultaneously treated with DEXA and HPKS, 3 times more B-ALL cells died (about 35%) (Figure 1E), almost equivalent to B-ALL cells without support. Interestingly, these results were very similar when PKC was inhibited with ENZA (Supplementary Figure S1A,B). This experiment not only suggests that the double treatment increases the leukemic cell susceptibility to therapy, but even more important, that in co-culture conditions, the role of PKC in MSC is more relevant that its counterpart in B-ALL cells. Together, these results confirm that PKC inhibition not only has a direct effect on leukemic cell viability [16], but also an indirect effect, MSC-mediated, in B-ALL cell sensitization to chemotherapy.

### 2.2. NF-κB and Akt Signalling Are Increased in MSC after the Co-Culture with B-ALL Cells

**Figure 2:**
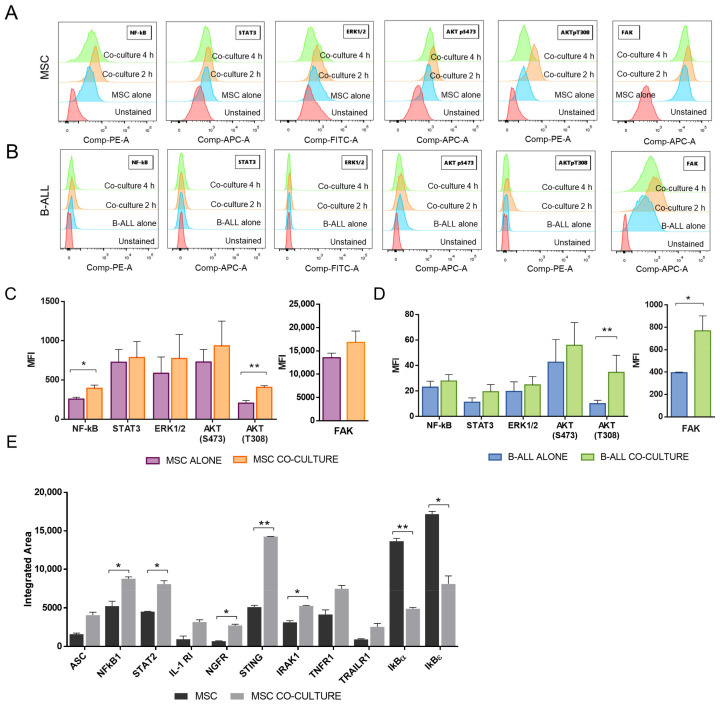
Direct contact between B-ALL cells and MSC involves mainly the activation of AKT and NF-κB signaling in the stromal support. (**A**, **B**) Changes in the activation of AKT, FAK, STAT3, NF-κB, and ERK in MSC (**A**) and B-ALL cell (**B**) populations. The phosphorylation status of the mentioned molecules was evaluated by flow cytometry. The co-cultures were established for 2 or 4 h, then non-adherent B-ALL cells were removed, and co-cultures were trypsinized, fixed, permeabilized, and incubated with phospho-specific antibodies. (**C**, **D**) Quantification of the activation of AKT, FAK, STAT3, NF-κB, and ERK, measured as MFI in MSC (**C**) and B-ALL cells (**D**) after 2 h of co-culture. Data correspond to three independent experiments. (**E**) Changes in the expression of molecules involved in NF-κB signaling. Protein extracts of MSC were obtained from MSC alone or in co-culture for 2 h with B-ALL cells; lysates were incubated in microarrays in order to evaluate 41 different molecules. Quantification of the dot density in the microarray is shown. In the graph, the molecules that increased or decreased by more than 50% are depicted. Data are from one experiment in duplicate and expressed as mean ± SEM (*p* values: Non-parametric one-way ANOVA (C y D) * *p* < 0.05, ** *p* < 0.01; and multiple *t*-test (**E**) * *p* < 0.05, ** *p* < 0.01).

The protective effect of MSC in drug treatment is dependent on cell adhesion and integrin signaling through the activation of several pathways, including PI3K/Akt, MAPK, and NF-κB [28]. We proceeded to evaluate these signaling pathways in MSC, and as can be seen in Figure 2A, NF-κB and Akt (pT308) were up-regulated in MSC after co-culturing with B-ALL cells. No statistically significant differences in the expression of STAT3, Akt (S473), or Erk1/2 were observed in MSC after co-cultivation (Figure 2C). Although FAK expression was not significantly altered in MSC after the co-culture period, it was increased in B-ALL cells (Figure 2B); FAK signaling in B-ALL cells was increased in the co-culture and sustained during the 4 h period studied (Figure 2B). Nevertheless, FAK expression in MSC was much higher than in B-ALL cells, as can be appreciated by comparing the scale bars. Additionally, an increase in AKT (pT308) was revealed in B-ALL cells (Figure 2D). The increased signaling observed in MSC was rapid and transient since it was detected 2 h after cell-to-cell contact but was reduced to basal levels in MSC 2 h later (Figure 2A). 

To further study the MSC-associated cell signaling involved in the protective mechanism of leukemia cells, we measured the expression of 41 proteins whose activity is associated with NF-κB signaling. The microarray analysis showed the activation of this pathway in MSC after B-ALL binding, with an increased expression of at least twice in NF-κB1, a difference that was statistically significant (Figure 2E). In addition, IL-1R and IRAK that are linked to NF-κB signaling were up-regulated in the MSC in the co-cultures. Additionally, the IKBε and IKBα inhibitors of the NF-κB pathway were down-regulated in the co-cultured MSC by more than half (Figure 2E). This represents additional evidence of the activation of NF-κB signaling in MSC after B-ALL cell binding. This is in agreement with the phosphorylation of NF-κB that we have previously observed by Western blot in a leukemic niche with the B-ALL REH cell line [43].

### 2.3. Blocking of NF-κB, but Not Akt, Inhibited Adhesion of B-ALL Cells to MSC without Increasing Susceptibility to DEXA or VNC

**Figure 3:**
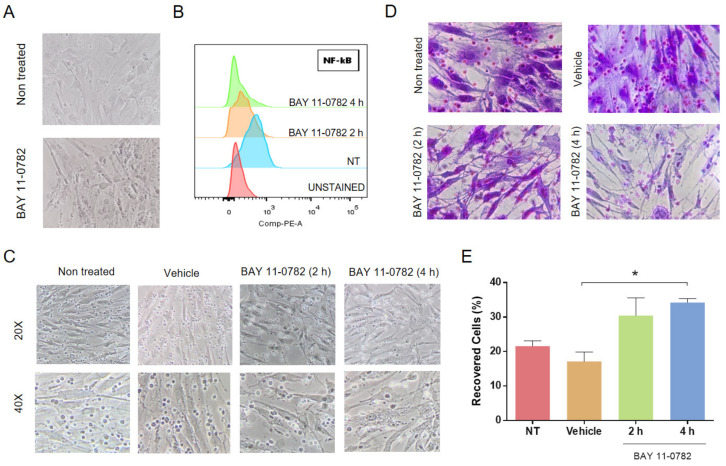
NF-κB inhibition in MSC alters the B-ALL cells’ adhesion. (**A**) Morphological changes in MSC after 4 h of treatment with 10 µM of BAY inhibitor. Representative microphotographs are shown at 40× magnification. (**B**) Effect of the BAY inhibitor on the phosphorylation of NF-κB was evaluated by flow cytometry after fixation and permeabilization. Non-treated (NT, blue histogram) MSC and unstained cells were used for comparisons (red histogram); orange and green histograms show the phosphorylation of NF-κB after BAY treatment after 2 and 4 h, respectively. (**C**) MSC morphological changes and B-ALL cell adhesion capacity evaluation after NF-κB inhibition in MSC. MSCs were pre-treated with BAY as indicated, and 2 h after, B-ALL cells were co-cultured on mesenchymal support for 6 h. Non-adherent leukemic cells were removed and counted. Microphotographs at 20 and 40× magnification are shown. (**D**) Co-cultures after removing non-adherent B-ALL cells and staining with crystal violet. Representative microphotographs are shown at 40× magnification. (**E**) Quantification of non-adherent B-ALL after the treatments described. The percentage of recovered cells was calculated using a Neubauer chamber based on the input of leukemic cells and the number of non-adherent B-ALL cells recovered after washing with PBS 1X. Data are expressed as mean ± SEM (*p* values: Non parametric one-way ANOVA. * *p* < 0.05) from two independent experiments in triplicate.

To find a functional connection between PKC and NF-κB, we assessed if, by directly inhibiting the transcription factor NF-κB, we could abolish the protective effect of MSC. First, we have verified NF-κB inhibition by BAY11-0782 (BAY), an inhibitor of IkBα phosphorylation and hence of its proteasomal degradation. As can be seen in Figure 3A, a 4 h treatment with BAY produced some changes in the morphology of MSC with the extrusion of rounded and opaque content; a 2 h treatment with this inhibitor reduced NF-κB activity to 50%, while a 4 h treatment almost abolished it (Figure 3B). Next, we evaluated the effect of this inhibitor on B-ALL cell adhesion to MSC. As expected, both treatments (2 and 4 h) augmented B-ALL cell detachment (Figure 3C,D); also, MSC looked thinner, more elongated, with a more irregular cell contour (Figure 3C) and appeared less stained with an apparent reduction in extracellular matrix components (Figure 3D). The reduction in cell adhesion in BAY treated cells was about 50% compared to controls (Figure 3E). This effect was also true for another B-ALL patient sample (Supplementary Figure S2A–D). We also employed the AKT inhibitor GSK690693, an ATP competitive inhibitor, to treat the MSC and evaluate its effect on leukemic cell adhesion (Supplementary Figure S2E,F). Interestingly, B-ALL cell adhesion was not hindered by Akt inhibition, as seen previously with NF-κB or PKC inhibition.

**Figure 4:**
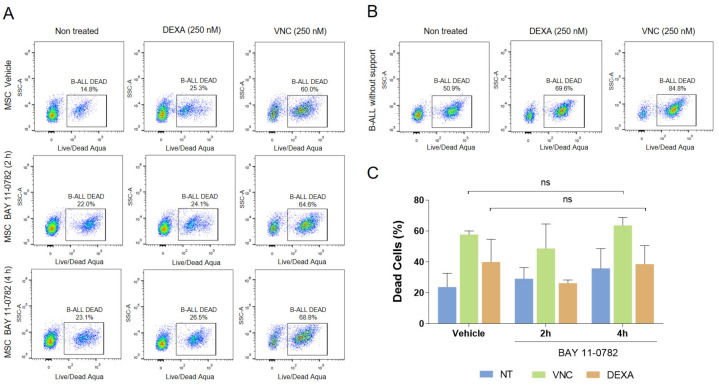
NF-κB inhibition slightly alters the support conferred to B-ALL cells by MSC without increasing the sensitivity of leukemic cells to chemotherapy. (**A**) Changes in B-ALL cell viability after inhibition of NF-κB in MSC support. MSC were treated for 2 or 4 h with BAY inhibitor (10 µM), and co-cultured with leukemic cells pre-treated or not (NT) with VNC or DEXA (250 nM) for 6 h. B-ALL cells viability was evaluated after 72 h of co-culture. LIVE/DEAD Aqua positive cells (in squares) correspond to dead cells and were defined based on the unlabeled counterpart. (**B**) B-ALL cells without support and treated with the respective drugs were also included as controls. (**C**) Quantification of B-ALL cells viability in co-cultures according to the indicated treatments. Data are expressed as mean ± SEM (*p* values: Non parametric one-way ANOVA. ns: Non-significant) from two independent experiments.

We then examined if NF-κB inhibition could also confer susceptibility to drug treatments. Unexpectedly, and contrarily to what we have previously observed with the inhibition of stromal PKC by HKPS or ENZA, we did not observe sensitization to DEXA or VNC after the pre-treatment of MSC with the NF-κB inhibitor BAY (Figure 4). Although B-ALL cells were protected from death by the MSC co-culture (compare Figure 4A,B), previous inhibition of NF-κB in MSC did not substantially change the effect of drug treatments (Figure 4C and Supplementary Figure S2C). This was true in two other B-ALL samples studied (Supplementary Figure S2D). 

### 2.4. Increased Sensitization to Drug Treatment Could also Be Dissociated from NF-κB and Akt Signalling

Taking into consideration that PKC and NF-κB inhibitors hindered the adherence of B-ALL cells to MSC, but only HKPS or ENZA increased the susceptibility to DEXA and VNC, we studied if these inhibitors were capable to modify the cell binding-associated signaling described above (Figure 2A). For this purpose, we have pre-incubated MSC with ENZA or the HKPS peptide for 2 h and then co-cultures with B-ALL cells were established for two additional hours. None of the inhibitors reduced the signaling of FAK, Akt, or NF-κB in MSC (Supplementary Figure S3A). On the contrary, a small increase in FAK with the HKPS (but not with ENZA) treatment was observed in MSC. Otherwise, no changes associated with the treatment of PKC inhibitors were observed in B-ALL cells (Supplementary Figure S3B).

To further shed light on the signaling involved in the sensitization induced by PKC inhibition, we thought that another complementary approach to the problem would be to test the changes in signaling induced by drug treatment and then study effect of HKPS on this signaling. HKPS was selected since the chimeric peptide showed a higher effect than ENZA on B-ALL cells sensitization to chemotherapy. The molecules involved in NF-κB signaling and induced by the co-culture were almost unaffected by DEXA or VNC treatment (Supplementary Figure S4A). Additionally, HKPS had a minimal effect on these signaling molecules (Supplementary Figure S4B,C). These results suggested that other signaling molecules and/or other cellular mechanisms must be considered and that the increased susceptibility of B-ALL cells is not only due to cell detachment.

### 2.5. Evaluation of Other Kinases That Could Be Involved in Drug Protection

**Figure 5:**
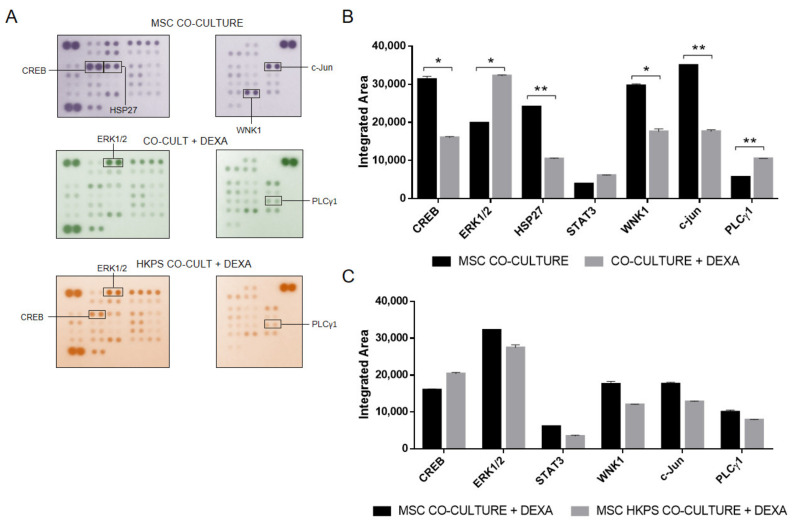
Sensitization to chemotherapy by HKPS could involve other kinases. (**A**) Changes in the phosphorylation profile of MSC after treatment with DEXA. Co-cultures were established with B-ALL cells and treated or not with DEXA (500 nM) for 2 h. In addition, MSC were pre-treated with HKPS for 2 h, then co-cultures were established for an additional 2 h in the presence of DEXA. After removal of B-ALL cells, MSC were lysed, and 350 ug of protein were incubated in a proteome array. The molecules that changed more than 50% are indicated in a square, as well as those that were inhibited by HKPS. (**B**) Quantification of the phosphorylation status of the kinases with the highest variation after DEXA treatment. The integrated area was determined based on the intensity of each dot. (**C**) Quantification of the phosphorylation status of the kinases with the highest variation after HKPS and DEXA treatments. In the column graphs, data are expressed as mean ± SEM (*p* values: Multiple *t*-test * *p* < 0.05, ** *p* < 0.01). Data are from one experiment in duplicate.

Similarly, we followed the same approach for the evaluation of 43 other phosphokinases that could participate in drug resistance induction by MSC. First, we evaluated them in the co-culture system (Figure 5A,B) and then after HKPS pre-treatment of the MSC support (Figure 5C). DEXA treatment induced an important increase in the phosphorylation of Erk1/2 (60%) and PLCγ1 (80%) and a reduction of CREB (50%), HSP27 (57%), WNK1 (40%), and c-Jun (50%) (Figure 5A,B). The changes in Erk1/2, PLCγ1, and CREB were prevented by prior treatment of MSC with HKPS (Figure 5C), suggesting that these three molecules could be responsible for the susceptibility induced by HKPS and that they could be connected to PKC signaling. 

### 2.6. B-ALL ABC Transporters Are Indirectly Affected by the MSC Support

**Figure 6:**
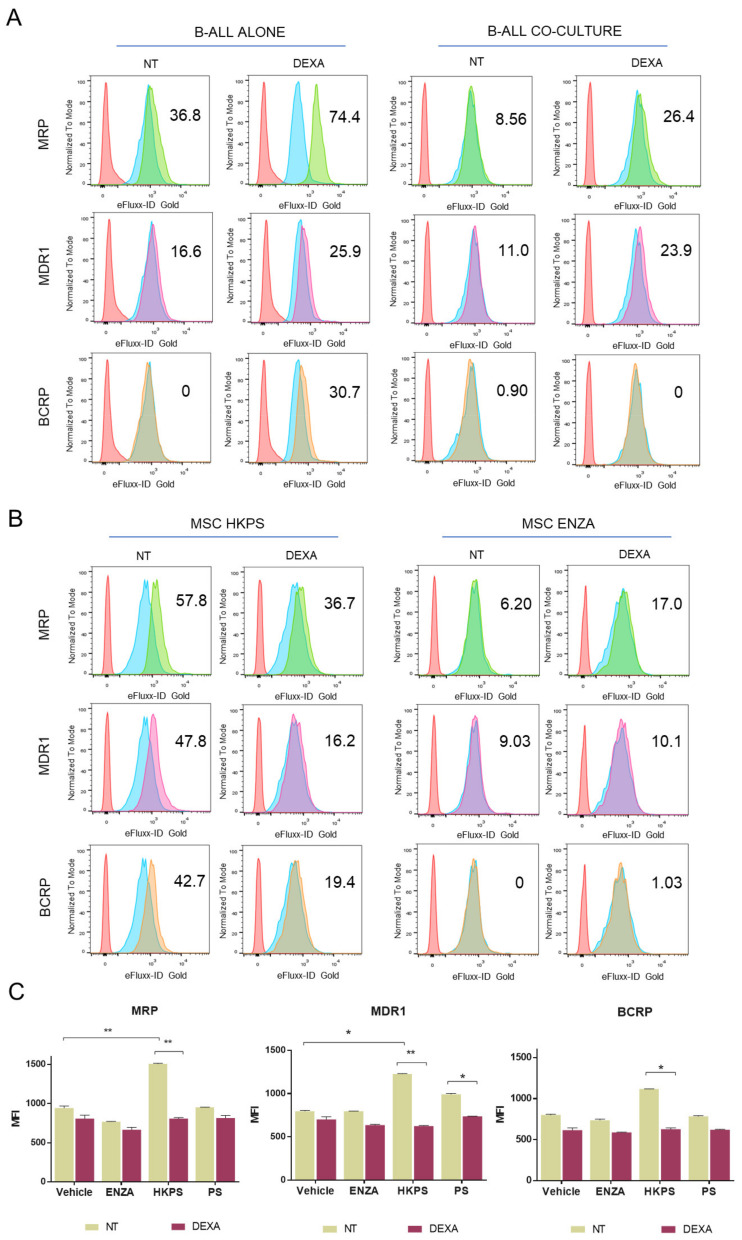
The co-culture with MSC induces changes in ABC transporters’ activity in B-ALL cells, and PKC inhibition with HKPS increases this effect. (**A**) Influence of the mesenchymal support on MDR1, MRP, and BCRP activity in leukemic cells. Co-cultures were treated with 250 nM of DEXA for 72 h, then both MSC and adherent B-ALL cells were incubated with specific inhibitors for each transporter. Comparatively, leukemic cells in suspension were treated with the chemotherapeutic agent in similar time and concentration conditions. The transporter’s activity was evaluated by flow cytometry, it is expressed as MAF (multidrug resistance activity factor), based on the MFI values in the presence of the respective inhibitor and the basal activity without inhibitor. Red histograms correspond to unstained cells, blue to the vehicle (1.25% DMSO) (basal activity), and green, violet, and orange to cells treated with the inhibitors (MK-571, Verapamil, and Novobiocin, respectively). (**B**) Effect of PKC inhibition on drug efflux by ABC transporters. MSC were pre-treated with HKPS (40 µM) or ENZA (20 µM) for 2 h, and co-cultures were established for additional 72 h in the presence of DEXA (250 nM). The transporter’s activity was determined by flow cytometry as indicated before. (**C**) Quantification of the ABC transporters activity in B-ALL cells after PKC inhibition in MSC. As control, the PS peptide (40 µM) was used since the hydrophobic HK sequence could interfere with the transporters activity. Data are expressed as mean ± SEM (*p* values: two-way ANOVA. * *p* < 0.05. ** *p* < 0.01) from two independent experiments; NT: non- treated.

Since susceptibility to drug treatment was not only dependent on cell adhesion, we looked for other B-ALL cell modifications that could occur after co-cultivation with MSC. In particular, drug extrusion has been described as a chemoresistance mechanism, in which leukemic cells increase the activity of the ATP-binding cassette (ABC) transporters in the presence of MSC [44]. We employed Verapamil, Novobiocin, and MK-571, three specific inhibitors of MDR1, BCRP, and MRP ABC transporters in order to evaluate their activity. First, ABC transporters’ activity was evaluated in B-ALL cells alone or treated with DEXA and in B-ALL cells co-cultured with MSC, and then in co-cultures after previous inhibition of MSC PKC with ENZA or HKPS. To be sure that the effect of HKPS on ABC transporters was not due to leukemic cell detachment, B-ALL cells in suspension were washed out and transporter activity was measured in B-ALL cells bound to MSC. As expected, in B-ALL cells without support, DEXA induced an increase in the activity of MDR1, BCRP, and MRP (Figure 6A left panels). The presence of MSC reduced this effect in adherent B-ALL cells treated with DEXA; however, the activity of MDR1 and MRP1 (but not BCRP) remained higher in the presence of DEXA in comparison to the non-treated control (Figure 6A right panels). This suggests that MSC modulates changes in the activity of some of these transporters in B-ALL cells, reducing the effect caused by DEXA. Remarkably, pre-treatment of MSC with HKPS induced a decrease in the activity of MDR1, MRP, and BCRP transporters in B-ALL cells (Figure 6B left panels and Figure 6C), but these effects were not observed with the pre-treatment of MSC with ENZA (Figure 6B right panels). Unexpectedly, PS induced a slight but significant effect in the MDR1 transporter. These results indicated that there is an indirect effect caused by HKPS treatment of MSC, not related to its cell-binding inhibitory capacity, which can render leukemic cells more susceptible to chemotherapy.

## 3. Discussion

The approach to overcome tumor heterogeneity by deciphering the components of the BM niche responsible for leukemic cells survival, chemoresistance, and disease progression seems to be a valid strategy to control leukemia [45,46]. The protective effect of MSC and the role that PKC-β has in chemoresistance were duly demonstrated in a murine experimental model and in in vitro co-cultures of human primary cells of CLL [17]. Importantly, this shielding effect was extended to different CLL treatments and B cell malignancies (ALL and mantle cell lymphoma). Additionally, we have shown that, in spite of the existence of diverse mechanisms of cell adhesion [22,39,47,48], blocking B-ALL cell interactions with MSC by inhibiting MSC PKC activity has a drug sensitization effect in B-ALL cells [16]. Interestingly, this effect on leukemic cells seems to be independent of their diverse genetic defect and the chemotherapeutic agent employed, as we have shown before for DEXA [16] and here for VNC and MTX, suggesting that the strategy could have widespread use. Additionally, the simultaneous treatment of the co-cultures with DEXA and any of the PKC inhibitors used had an important effect on B-ALL cell viability, practically abolishing the MSC protective effect.

To investigate the mechanisms responsible for this loss of supportive signals and increased sensitivity, we first studied the PKC-associated signaling that could be involved in this process. Yet, PKC function is very complex, mediating different cellular physiological processes by the activation of several signaling pathways, including JAK/STAT, JNK/c-Jun, p38, MEK/ERK, and NF-κB, among others [49]. We found that the co-culture of B-ALL cells with MSC induced significant activation of Akt and NF-κB signaling in MSC; the participation of the latter was further confirmed by the up-regulation of NF-κB1 and down-regulation of the inhibitors IKBε and IKBα of this signaling pathway. This is in line with our previous experiments showing NF-κB phosphorylation in an in vitro leukemic niche [43] and the well-documented key role of NF-κB in MSC support [38]. 

Since chemoresistance is also strongly associated with leukemic cell binding to stroma and PKC activity in MSC [16,17], we explored the relationship between PKC and NF-κB and their role in this process using two strategies. First, we studied the effect of DEXA and VNC treatment in NF-κB signaling in MSC in co-culture with B-ALL cells and how this signaling was affected by the pre-treatment of MSC with HKPS. Second, we have used inhibitors of the signaling pathways that were up-regulated in MSC by B-ALL cell binding and studied their effect on cell binding and B-ALL cell sensitization to drug treatment.

Minimal changes were observed in NF-κB-associated signaling when co-cultures were treated with DEXA or VINC. Additionally, HKPS pre-treatment of MSC did not substantially modify any of the molecules studied. We then used inhibitors of Akt and NF-κB signaling to study their involvement in cell adhesion. Interestingly, only the inhibition of NF-κB induced a detachment of B-ALL cells from MSC, suggesting that it could be the molecular connection with PKC and part of the mechanism of drug sensitization. Because PKC inhibition induced B-ALL sensitization to DEXA [16], VNC, and MTX (this study), through a decreased cell-adhesion-mediated mechanism, we evaluated drug sensitization in the presence of the BAY inhibitor of NF-κB. Surprisingly, no important differences were observed in the survival of B-ALL cells after DEXA or VNC treatment. Therefore, increased sensitization to drug treatment could be dissociated from NF-κB and Akt signaling. 

These results suggested to us that (1) HKPS- or ENZA-induced susceptibility is independent of NF-κB and Akt signaling, and therefore other signaling pathways must be considered and (2) the increased susceptibility reached by altering the mesenchymal support with these PKC inhibitors is not only due to cell detachment and therefore other cellular mechanisms must be considered. In fact, NF-κB inhibition was sufficient to reduce ALL and CLL cell survival in monoculture but was totally inadequate to induce leukemic cell death in the presence of stromal support [37,50], implying that alternative mechanisms operate together in leukemic cell protection.

Depending on the drug employed, chemotherapy can also induce changes in the microenvironment [8,41]. The microarray analysis showed that from the 41 phosphokinases evaluated, Erk1/2 and PLCγ1 were up-regulated (>50%) by DEXA treatment of the co-cultures, while CREB, HSP27 WNK1, and c-Jun were down-regulated (>50%). Importantly, of these six molecules, only the phosphorylation of Erk1/2, PLCγ1, and CREB was modulated by HKPS pre-treatment of MSC. Of note, signaling pathways that affect chemoresistance have been mainly studied in B-ALL cells [29,51] and not in the mesenchymal support. Therefore, Erk1/2, PLCγ1, and CREB modulation in MSC could become new molecular targets or signaling pathways to be further explored. The precise participation in drug sensitization of PLCγ1, Erk1/2, and CREB and their eventual interaction deserves additional research.

In several instances of hematological neoplasia, the interaction between MSC and leukemic cells induces an over-activation of ABC transporters, in typically quiescent and drug-resistant cell populations [44]. When the ABC transporter’s function was assessed, after DEXA treatment of B-ALL cells in a co-culture system with HKPS-pretreated MSC, we found a decrease in the MDR1, BCRP, and MRP activities in B-ALL cells. This effect was indirect since MSC were the cells exposed to the HKPS peptide, while the effect on ABC transporter activity was observed in leukemic cells. Importantly, this effect was observed in B-ALL cells bound to MSC, suggesting that it can be separated from the effect that HKPS has on cell binding and could be due to a diminished supportive capacity of MSC. In this sense, we have observed that ALDH activity (having a cytoprotective role) was reduced in HKPS-pretreated MSC (not shown). 

All in all, we have shown that sensitization to different drugs could be reached by interfering with the functional supportive niche induced by interactions of B-ALL cells with MSC. In particular cell-binding inhibition appears to be one strategy, but in some cases, simultaneous alteration of particular signaling pathways and/or other cellular protective mechanisms are needed. The inhibition of NF-κB, a transcription factor associated with adhesion molecule signaling and in fact necessary for B-ALL cell binding to MSC, as shown here, was not able to induce sensitization to DEXA or VINC, and therefore seems not to be the exclusive molecular connection through which stromal PKC protects B-ALL cells. On the other hand, PKC inhibition, particularly by the chimeric HKPS peptide, was very effective in inhibiting both cell binding and decreasing ABC transporter activity, and consequently inducing drug sensitization. The fact that HKPS showed a higher indirect sensitization effect on drug treatment than ENZA and that it was the only compound capable to inhibit the activity of MDR1, MRP, and BCRP transporters suggests that it could be a valuable tool for increasing cytotoxicity of B-ALL cells. Previously, we have shown that HKPS was also more cytotoxic to B-ALL cells than the strong and more unspecific PKC inhibitor, Staurosporine [16], showing even more its eventual therapeutic effectiveness. Of interest, Erk1/2, PLCγ1, and CREB, molecules altered by dexamethasone treatment and whose alterations were reduced by previous HKPS incubation of MSC, should be the subject of further study since they seem to be linked to PKC and to have a particular role in sensitization. Importantly, the decreased activity of ABC transporters induced by DEXA treatment of B-ALL cells after HKPS pre-treatment of MSC represents a novel mechanism of sensitization induced by PKC inhibition. B-ALL cells have different strategies to subvert drug treatment; therefore, various and diverse approaches should be envisaged to induce B-ALL cell death. 

## 4. Materials and Methods

### 4.1. B-ALL Cell Samples and MSC Isolation and Characterization

B-ALL cells were isolated from BM aspirates of newly diagnosed pediatric patients that had not received any treatment, and with a blast infiltration higher than 80%. Characterization of patients was carried out in the pediatric Oncohematology Service of the Fundación Hospital de la Misericordia, following the WHO criteria and the cell immunotypification was performed in a FACScanto II cytometer (Becton Dickinson Biosciences, San Jose, CA, USA), according to the Euroflow panel recommendations (www.euroflow.org. accessed on 28 February 2019). Infinicyt Software, v. 2.0 (Cytognos SL, Salamanca, Spain) was employed in data analysis. For experimental use, the mononuclear cells (MNC) were isolated by centrifugation gradient with Ficoll–Hypaque (Sigma-Aldrich, St. Louis, MO, USA). Cells were used immediately or frozen at −70 °C until use. Preliminary experiments showed that there were no differences in viability after thawing. In total, 12 B-ALL pediatric patient samples were used for the experiments.

MSC (*n* = 4) were obtained from healthy pediatric patients that consulted for orthopedic events. BM aspirates were collected in tubes with PBS 1X and 0.25% EDTA. The MNC were isolated by gradient centrifugation with Ficoll–Hypaque (Sigma-Aldrich, St. Louis, MO, USA). MNC were seeded in plastic cell culture flasks, and after 24 h, non-adherent MNC were removed by washing with PBS 1X. Adherent MSC were characterized by flow cytometry after reaching 90% confluence by the expression of CD44 (Clone G44-26, BD Pharmigen, San Jose, CA, USA) CD90 (clone F15-42-1, Abcam, Cambridge, MA, USA), CD105 (clone 43A4E1, Miltenyi Biotech, Bergisch Gladbach, Germany), and CD73 (clone AD2, BD Pharmingen, San Jose, CA, USA) in the absence of CD45 (clone 2D1, BD Biosciences, San Jose, CA, USA) and CD34 (clone 581, BD Pharmingen, San Jose, CA, USA) markers. MSC were also characterized by their multilineage differentiation capacity assays according to ISCT criteria [52] and as previously reported [16]. MSC were frozen in several vials for its subsequent use and employed between passages 3–5. The same sample and same passage was used for equivalent/similar experiments. 

In both cases, the parents who authorized the use of the bone marrow samples signed the informed consent previously approved by the ethical committees of the HOMI and Facultad de Medicina, UNAL (007-080-17, 11 May 2017). Ethical aspects concerning human experimentation followed the principles of the Declaration of Helsinki [53].

### 4.2. Inhibitors of Signalling Molecules Used

For PKC inhibition, conventional and novel compounds were used. Enzastaurin (ENZA) (Sigma-Aldrich, St. Louis, MO, USA), a commercial inhibitor of PKCβ, was employed in parallel with the HKPS chimeric peptide, an inhibitor of classical PKC isoforms (cPKC) previously described by us [16,54]. This peptide was synthesized by the SPPS-Fmoc/tBu method [55], purified (with >95% purity), and characterized by RP-HPLC and MALDI-TOF mass spectrometry, respectively. The peptides HK (the hydrophobic cell permeable sequence) and PS (the regulatory natural PKC pseudosubstrate sequence) were used as controls. Stock solutions of the peptides were prepared in DMSO and diluted with PBS to use in the different experiments; DMSO concentration was always below 0.1%. For NF-κB and Akt inhibition, the compounds BAY11-7084 (BAY) (Abcam, Cambridge, UK), and GSK 690693 (Abcam, Cambridge, UK) were used, respectively. Treatments with these inhibitors were administered in incomplete culture media at the concentrations indicated in each experiment. 

### 4.3. Cell Adhesion Assays

The functional assays of cell adhesion were performed in 96-well plates. Furthermore, 1 × 10^4^ MSC were seeded in 100 µL of IMDM medium (GIBCO-Life Technologies, Grand Island, NY, USA) supplemented with 10% of FBS (GIBCO-Life Technologies, Grand Island, NY, USA), 1% sodium pyruvate (Corning, Fisher Scientific, Manassas, VA, USA), and 1% of non-essential aminoacids (LONZA, Walkersville, MD, USA). After 24 h, MSC were treated with the PKC inhibitors ENZA (Sigma-Aldrich, St. Louis, MO, USA) (20 µM), HKPS (40 µM), or the NF-κB inhibitor BAY (Abcam, Cambridge, UK) (10 µM) for 2 or 4 h in incomplete medium. The inhibitors were removed by washing with PBS 1X, and co-cultures were established by seeding 5 × 10^4^ B-ALL cells on the mesenchymal support. After 6 h of culture, non-adherent B-ALL cells were collected by gently washing with PBS 1X and counted manually in a Neubauer chamber. The percentage of recovered leukemic cells was calculated based on the initial input of B-ALL cells and the non-adherent cells in the supernatant. The co-cultures were fixed with absolute methanol and stained with 0.5% crystal violet. Microphotographs were acquired in an inverted microscope (Eclipse Model TS-100, Nikon, Konan, Minato-ku, Tokyo, Japan). 

### 4.4. Phosphorylation Evaluation by Flow Cytometry

The intracellular phosphorylation status of NF-kB, FAK, AKT, STAT3, and ERK1/2 was evaluated by flow cytometry. After 2 or 4 h of co-culture of MSC and B-ALL cells, non-adherent leukemic cells were removed by washing with PBS 1X. The remaining MSC and B-ALL adherent cells were trypsinized with 0.25% Trypsin and 1 mM EDTA and fixed with Fix Solution Buffer (Biolegend, San Diego, CA, USA) for 30 min. Cell identification was performed by flow cytometry, based on FSC and SSC. Intracellular staining Perm Wash Buffer 1X (Biolegend, San Diego, CA, USA) was added to each tube and after 20 min incubation, cells were washed by centrifugation rounds at 600× *g* every 5 min. MSC and B-ALL cells were incubated according to the manufacturer recommendation with 20 µL of each antibody per test, the following anti-human monoclonal antibodies were employed: PE Mouse Anti-Akt (pT308) (Clone J1-223.371, BD Phosflow, San Jose, CA, USA), Alexa Fluor 647 Mouse anti-Akt (pS473) (Clone M89-61, BD Phosflow, San Jose, CA, USA), Alexa Fluor 488 Mouse Anti-ERK1/2 (pT202/pY204) (Clone 20A, BD Phosflow, San Jose, CA, USA), Alexa Fluor 647 Mouse Anti-Stat3 (pY705) (Clone 4/P-STAT3, BD Phosflow, San Jose, CA) PE Mouse anti-FAK (pS910) (Clone K73-480, BD Phosflow, San Jose, CA, USA), and PE Mouse anti-NF-κB p65 (pS529) (Clone K10-895.12.50, BD Phosflow, San Jose, CA, USA). Data acquisition was performed in a FACSAria III cytometer (Becton Dickinson Biosciences, San Jose, CA, USA) and the analysis was performed using the FlowJo software (v10.0, BD, FlowJo, LLC, Ashland, OR, USA).

### 4.5. ABC Transporters Activity

The EFLUXX-ID Gold multidrug resistance assay kit (ENZO Life Sciences, Farmingdale, NY, USA) was employed to determine the activity of the MDR1, MRP1, and ABCG2 transporters. Briefly, after treatments, co-cultures of MSC and B-ALL cells were trypsinized and incubated separately with the inhibitors verapamil, MK-571, or novobiocin for 5 min. DMSO was used as control of the basal activity. The cells were incubated with the Gold Dye reactive provided in the Kit and the Mouse anti-human CD19 (clone SJ25C1, BD Pharmingen, San Jose, CA, USA) and mouse anti-human CD73 (clone AD2, BD Pharmingen, San Jose, CA, USA) antibodies for 30 min. Propidium iodide supplied in the kit was added at the end of the incubation period in order to assess the transporters activity in the viable leukemic cell population. Immediately, the transporter’s activity was analyzed by flow cytometry. The analysis was done using the FlowJo Software (v10.0, BD, FlowJo, LLC, Ashland, OR, USA), and with the mean fluorescence intensity (MFI) values, the multidrug resistance activity factor (MAF) was calculated following the instructions provided by the manufacturer. 

### 4.6. Phospho Kinase Activity and NF-κB Pathway

The activation of different phospho kinases and the expression of other molecules involved in the regulation of the NF-κB signaling was determined using de Proteome Profiler Human Phospho-Kinase Array (ARY003B, R&D Systems, Minneapolis, MN, USA) and the Proteome Profiler Human NF-κB Pathway Array (ARY029, R&D Systems, Minneapolis, MN, USA) following the instructions of the manufacturer. Briefly, 1.2 × 10^6^ MSC were seeded until confluence in culture flasks of 75 cm^2^ with the IMDM medium supplemented with FBS 10%, sodium pyruvate, and non-essential amino acids. MSC were treated with 40 µM of HKPS for 2 h, and co-cultures with B-ALL cells were established for 2 additional hours. In some cases, the co-cultures were treated with DEXA (Tocris Biosciences, Bristol, UK) or VINC (Tocris Biosciences, Bristol, UK) (500 nM). B-ALL cells were removed after several washes with PBS 1X and PBS 1X + EDTA. 

MSC were lysed for 30 min at 4 °C with the buffer provided in the kit. The protein was quantified by the BCA method (Thermo Fisher Scientific, Waltham, MA USA). The arrays were incubated with 200 or 350 ug of protein overnight at 4 °C. Membranes were incubated with the corresponding detection antibodies according to manufacturer indications, and the membranes were exposed to film between 2 to 15 min, until the spots were easily and clearly appreciable. The photograph acquisition was done employing Gene Tool software (Syngene, Frederick, MD, USA) in a Gel Imagen System (GeneGenius, Syngene, CA, USA) and for the analysis of the dots with ImageJ software (National Institutes of Health, Bethesda, MD, USA), the integrated area for each molecule was determined using the macro, Protein Array Analyzer (V.1.1.c).

### 4.7. Drug Sensitivity in B-ALL Cells

Sensitivity of B-ALL cells to chemotherapy was evaluated assessing the leukemic cells viability after 3 days of co-culture with MSC. MSC were treated for 2 h with PKC inhibitors ENZA (20 µM) and HKPS (40 µM), the NF-κB inhibitor BAY (10 µM), or the vehicle DMSO. Leukemic cells were pre-treated with MTX (6.5 µM) or VINC (500 nM) for 6 h and then co-cultures were established for 72 h more. Co-cultures were trypsinized and double labelled with the anti-human CD19 antibody (clone SJ25C1, BD Pharmingen, San Jose, CA, USA) and the LIVE/DEAD Aqua reactive (Molecular Probes, Eugene, OR, USA). Cell viability was determined by flow cytometry and the data analysis was performed using FlowJo software (v10.0, BD, FlowJo, LLC, Ashland, OR, USA). 

For determining the simultaneous effect of chemotherapy and PKC inhibitors, leukemic cells were pre-treated for 6 h with DEXA (250 nM), HKPS (40 µM), ENZA (40 µM), or a combination of both; treatments were removed, and B-ALL cells were co-cultured with MSC for additional 24 h. In parallel, MSC and B-ALL cells were co-cultured and treated for 6 h with the combination of DEXA and the respective PKC inhibitor, treatments were then removed, and the cell viability was assessed after 24 h by flow cytometry as indicated above. As a control, B-ALL cells without support were included. Data analysis was performed using FlowJo software (v10.0, BD, FlowJo, LLC, Ashland, OR, USA).

### 4.8. Statistical Analysis

Data were expressed as the mean ± standard error of the mean (SEM). The phosphorylation status assessed by flow cytometry and the effect of PKC and NF- κB inhibition on B-ALL support were analyzed using non-parametric one-way ANOVA and the Kruskal–Wallis test. Comparisons of ABC transporters’ activity were performed using two-way ANOVA and microarrays analysis through the multiple *t*-test. Analyses and graphs were made using Prism version 8.0 software (GraphPad, San Diego, CA, USA) where *p* < 0.05 (*), *p* < 0.01 (**), and *p* < 0.001 (***) were considered to be statistically significant.

## Supplementary Materials

The following Figures are available online. Figure S1: B-ALL cell susceptibility to DEXA and ENZA in combined treatments; Figure S2: Effect of NF-κB and AKT inhibition on cell adhesion and sensitivity to chemotherapy in another sample of a B-ALL patient; Figure S3: Changes in FAK, AKT and NF-ΚB phosphorylation after inhibition of PKC; Figure S4: Effect of chemotherapy and HKPS on NF-κB signaling.

## Sample Availability

Samples of some PKC and ABC transporters inhibitors (as reference compounds) are available from the authors.
