# Supplementary material for: Sensitization to Drug Treatment in Precursor B-Cell Acute Lymphoblastic Leukemia Is Not Achieved by Stromal NF-κB Inhibition of Cell Adhesion but by Stromal PKC-Dependent Inhibition of ABC Transporters Activity
Source: Molecules. 2021 Sep 3;26(17):5366. doi: 10.3390/molecules26175366 (PMC8433757; doi:10.3390/molecules26175366)
Supplement: Supplementary file 1 [file molecules-26-05366-s001.zip › molecules-1328148-supplementary.pdf]

Sensitization to drug treatment in precursor B-cell acute lymphoblastic leukaemia is not achieved by stromal NF-κB inhibition of cell adhesion but by stromal PKC-dependent inhibition of ABC transporters activity

Paola Fernanda Ruiz-Aparicio[1], Gloria Inés Uribe[2,3], Adriana Linares-Ballesteros[3] and Jean-Paul Vernot[1,4,*].

SUPPLEMENTARY FIGURES

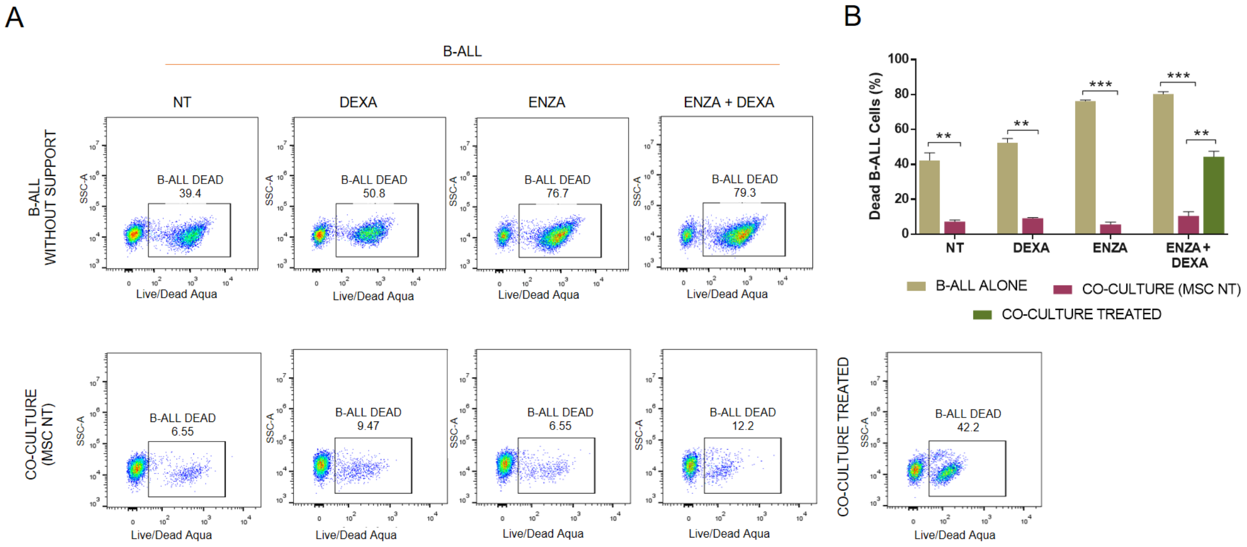

Suppl. Fig 1. B-ALL cell susceptibility to DEXA and ENZA in combined treatments. (**A**) Simultaneous effect of DEXA and ENZA on B-ALL cells viability. Leukemic cells were pre-treated for 6 h with DEXA (250 nM), ENZA (40 μM) or both, and then they were co-cultured for 24 h with non-treated MSC (CO-CULTURE (MSC NT)); also, co-cultures were established and then cells were treated with the combination of ENZA and DEXA (CO-CULTURE TREATED) as controls B-ALL cells without support were employed (B-ALL ALONE, upper panel). Cell viability was assessed by flow cytometry. A representative experiment is shown. (**B**) Percentages of B-ALL cells viability in the conditions indicated above. Data are from one experiment in duplicate. Data are expressed as mean ± SEM (p values: non parametric one-way ANOVA. $*p < 0.05$. $**p < 0.01$. $***p < 0.001$).

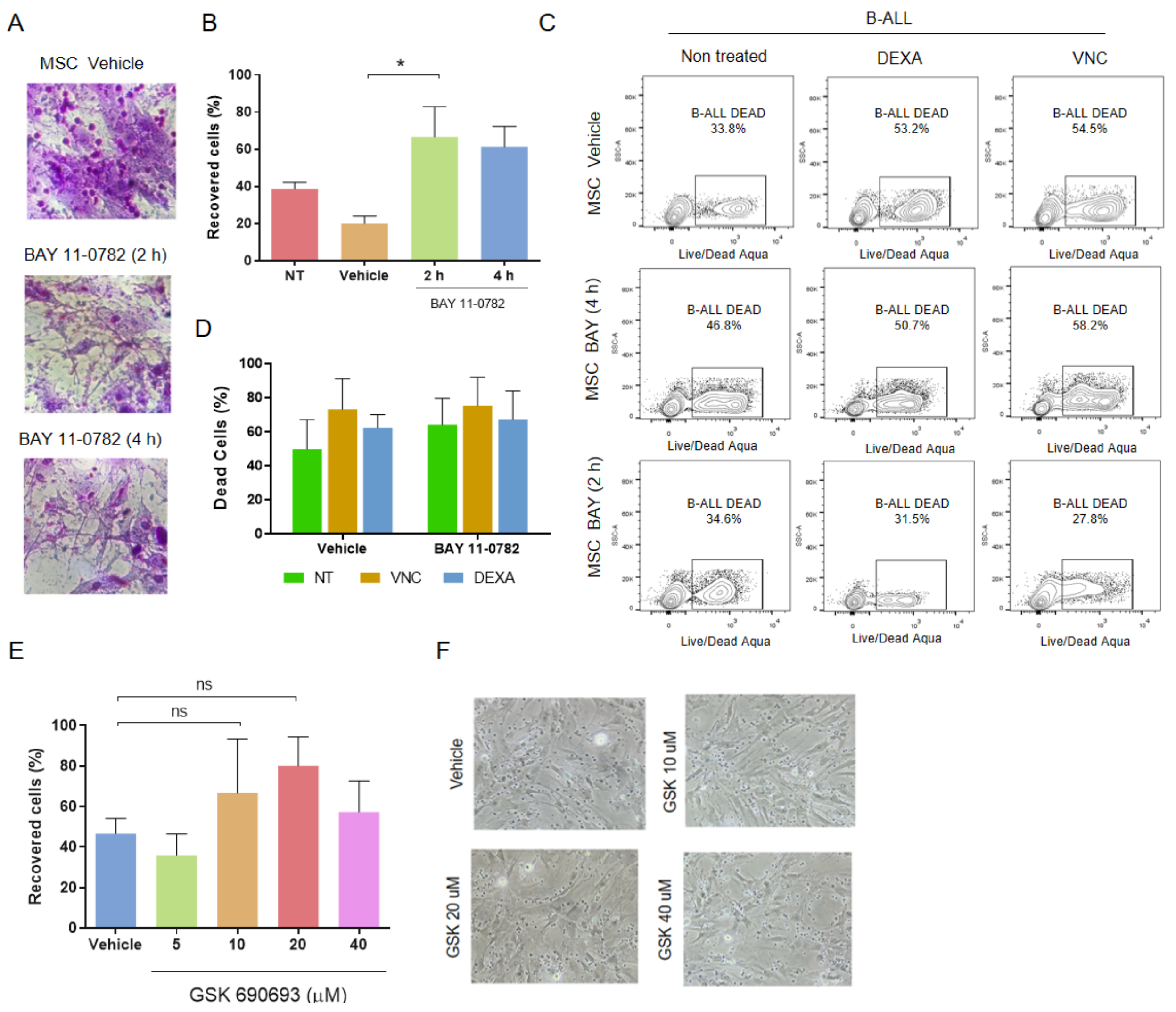

Suppl. Fig. 2 Effect of NF-κB and AKT inhibition on cell adhesion and sensitivity to chemotherapy in another sample of a B-ALL patient. Influence of NF-κB inhibition on B-ALL cell adhesion capacity to the MSC support. (**A**) Co-cultures with adherent B-ALL cells after NF-κB inhibition and crystal violet staining. Magnification 40x. (**B**) Quantification of B-ALL cell adhesion. MSC were pre-treated for 2 or 4 h with the BAY inhibitor (10 μM); co-cultures were established for additional 6 h, non-adherent leukemic cells were removed by washing and counted for assessing the percentage of recovery. (**C**) Influence of NF-κB inhibition on B-ALL cells susceptibility to drugs. MSC were pre-treated for 2 h with the BAY inhibitor, leukemic cells were treated for 6 h with DEXA or VNC (250 nM) and, after removal of the treatments, co-cultures were established for 72 h. Cell viability was assayed by flow cytometry after labelling co-cultures with CD19 and the LIVE/DEAD Aqua reagent. A representative experiment with primary cells from a B-ALL patient is shown. (**D**). Quantification of the percentage of B-ALL cells dead after NF-κB inhibition of the mesenchymal support and drug treatment as described before. Data from two independent experiments are shown. (**E**) Effect of AKT inhibition on leukemic cell adhesion. MSC were treated for 4 h with 5, 10, 20 or 40 μM of GSK inhibitor. Quantification of B-ALL cells recovered after washing. Percentage of non-adherent B-ALL cells were calculated based on the initial number of leukemic cells co-cultured with MSC. (**F**) Microphotographs at 20x magnification of co-cultures after treatment with GSK690693 (GSK) and removal of non-adherent leukemic cells.

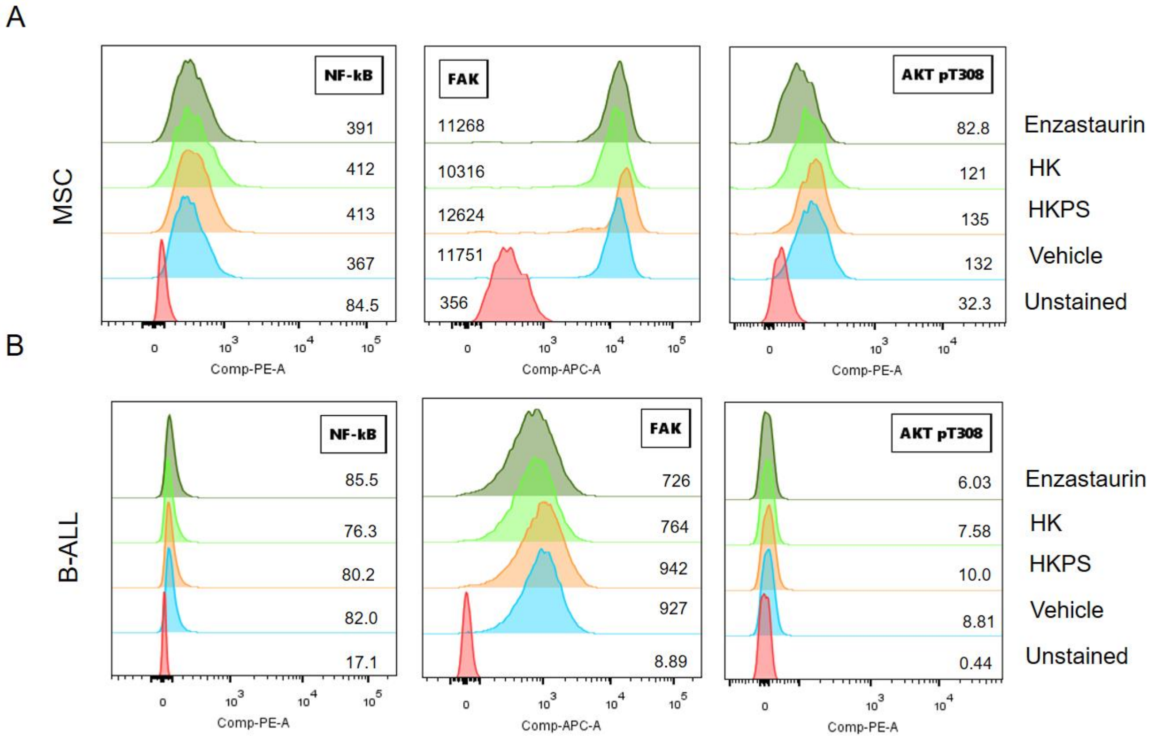

Suppl. Fig. 3 Changes in FAK, AKT and NF-KB phosphorylation after inhibition of PKC. (**A**) Effect of ENZA, HK and HKPS treatments in the mentioned signaling pathways. MSC were pre-treated for 2 h and co-cultures were established for additional 2 h. Non- adherent B-ALL cells were removed with PBS 1X, and co-cultures were fixed, permeabilized and incubated with the corresponding antibodies. Changes in cell signalling were determined in MSC (**A**) and leukemic cells (**B**). Numbers correspond to Mean Fluorescence Intensity (MFI) values.

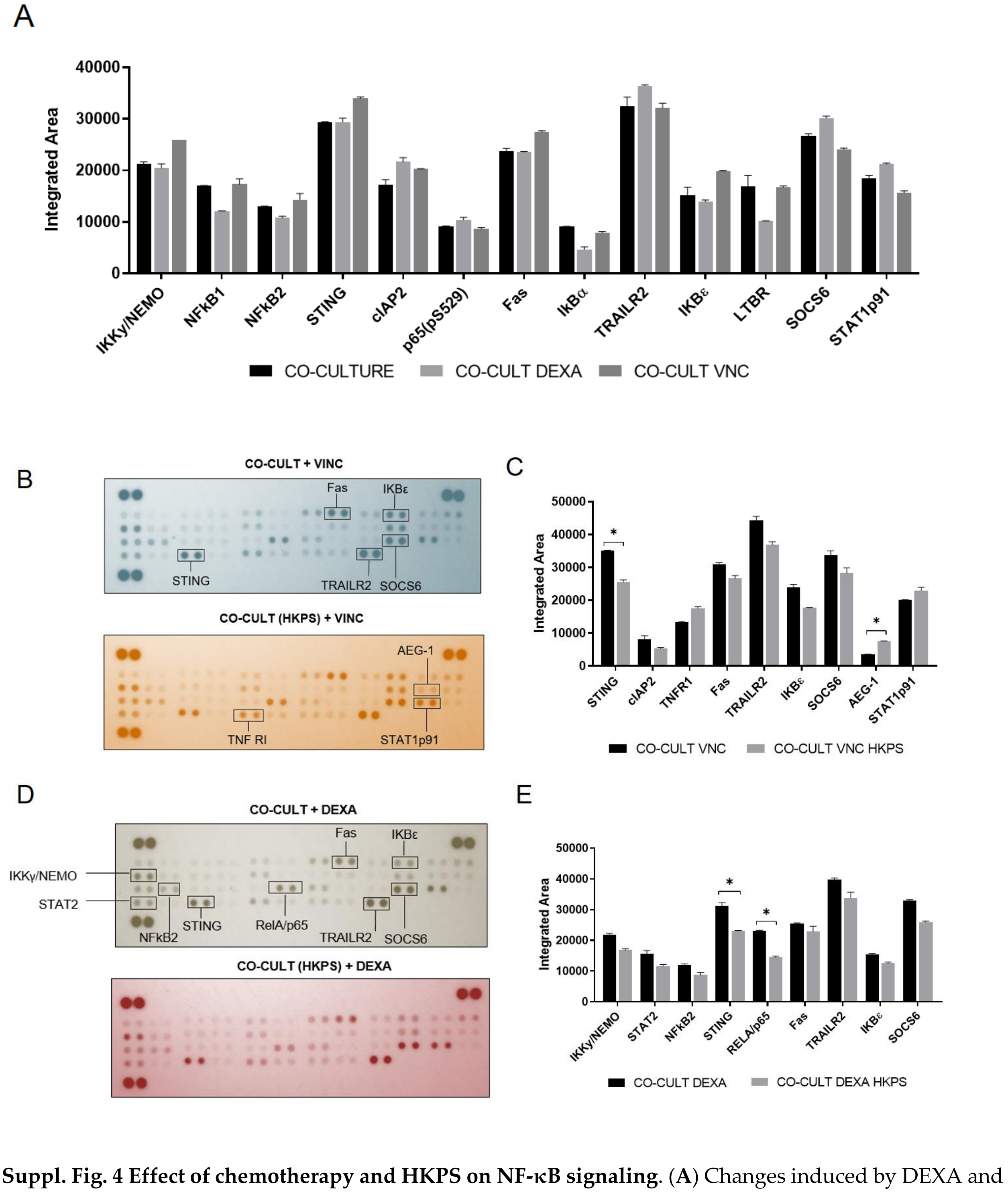

Suppl. Fig. 4 Effect of chemotherapy and HKPS on NF-κB signaling. (**A**) Changes induced by DEXA and VNC in the phosphorylation and expression of the different molecules involved in NF-κB activation or inhibition. Quantification of the dot density in the microarray. (**B, D**) Effect of the chemotherapy and HKPS treatment in the phosphorylation and expression of different molecules related to the NF-κB pathway. Protein extracts of MSC were obtained from MSC in co-culture for 2 h with B-ALL exposed to VNC (**B**) or DEXA (**D**). In some experiments, MSC were pre-treated for 2 h with HKPS prior to drug treatment, and cell lysates were incubated in microarrays. The molecules that were increased in each treatment are indicated in squares. (**C, E**) Quantification of the dot density in the microarray. Data are expressed as mean ± SEM (p values: multiple t test *$p < 0.05$). Data are from one experiment in duplicate.
